# Poricoic acid A attenuates renal fibrosis by inhibiting endoplasmic reticulum stress-mediated apoptosis

**DOI:** 10.1590/1414-431X2024e14249

**Published:** 2024-11-25

**Authors:** Hui Zhao, Tian Liu, Chang-E Yang, Yue-Huan Hu, Yan Niu, Sheng-Ping Lei, Lin Chen, Ming-Xia Zhang

**Affiliations:** 1 Clinical Experimental Center, Xi'an Engineering Technology Research Center for Cardiovascular Active Peptides, Northwest University Affiliated Xi'an International Medical Center Hospital, Xi'an, Shaanxi, China; 2 College of Biology, Pharmacy and Food Engineering, Shangluo University, Shangluo, Shaanxi, China

**Keywords:** Fibrosis, Poricoic acid A, Endoplasmic reticulum stress, Epithelial-to-mesenchymal transition, Apoptosis

## Abstract

Renal fibrosis is a common manifestation in the progression of chronic kidney disease (CKD) to kidney failure. Currently, there is no available therapy to prevent the progression of renal fibrosis. Poricoic acid A (PAA) isolated from *Poria cocos* shows notable antifibrotic effects. However, its potential mechanism is still unclear. This study aimed to evaluate the effects and the potential mechanisms of PAA against renal fibrosis. A mouse model of renal fibrosis was established using unilateral ureteral obstruction (UUO). We showed that PAA administration significantly alleviated renal lesions and collagen deposition in UUO mice. Mice with UUO resulted in epithelial-to-mesenchymal transition (EMT) and the activation of endoplasmic reticulum stress (ERS) in the renal tissues, while PAA treatment significantly inhibited EMT and ERS activation. Additionally, PAA markedly alleviated ERS-mediated apoptosis in UUO mice. Molecular docking results indicated that PAA stably combined to GRP78 and ATF4. In conclusion, these results demonstrated that PAA possesses a significant bioactivity against renal fibrosis and its treatment mechanism might be the inhibition of ERS-mediated apoptosis.

## Introduction

Chronic kidney disease (CKD) is one of the major diseases that seriously threaten human health with high morbidity and mortality worldwide (1). Renal fibrosis is a common pathological feature of CKD and is characterized by massive extracellular matrix (ECM) accumulation that causes the damage of renal tissue structure and loss of function (2,3). The renin-angiotensin system (RAS) inhibitors, including the angiotensin-converting enzyme inhibitors (ACEI) and angiotensin II receptor blockers (ARB), are currently the first-line drugs for the treatment of CKD. However, they are not exclusive targeted drugs for the therapy of renal fibrosis and have serious adverse reactions (4). It is, therefore, necessary to clarify the molecular mechanisms and develop new drugs for renal fibrosis.

The endoplasmic reticulum (ER) is an essential eukaryotic organelle for the synthesis, processing, and transport of protein and lipid. ER stress (ERS) is an essential protective response to adverse stimuli, which is caused by the unfolded protein response (UPR) in the endoplasmic reticulum (5). UPR is usually activated by three protein sensors, namely protein kinase RNA-like endoplasmic reticulum kinase (PERK), inositol-requiring protein 1 (IRE1), and activating transcription factor 6 (ATF6), which are respectively related to three signaling pathways (PERK-eIF2α-ATF4-CHOP, IRE1α-XBP1s, and ATF6) (6). Although ERS exerts an important protective role for the organism, sustained ERS may induce cellular apoptosis (7). Of note, emerging findings indicated that ERS-mediated apoptosis is associated with the pathogenesis and development of various kidney diseases (8,9).

**Figure 1:**
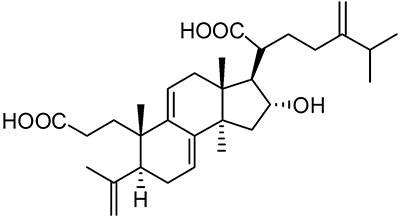
Chemical structure of poricoic acid A (PAA).

*Poria cocos,* belonging to polyporaceae family, is the sclerotium of *Poria cocos* (Schw.) Wolf, which is widely distributed in Anhui, Yunnan, Hubei, Hebei, Henan, and other provinces in China (10). As a well-known traditional Chinese medicine, *Poria cocos* (Chinese name: 茯苓) has been commonly used for its diuretic, tonic, and sedative effects (11). In recent years, growing evidence has shown that *Poria cocos* contains multiple tetracyclic triterpenoids, which exhibit significant effects against renal fibrosis (10,12). Among them, poricoic acid A (PAA, Figure 1) is the most representative component of *Poria cocos* and shows notable anti-fibrotic effects (13). PAA has been confirmed to exhibit a potent renoprotective effect by regulating multiple signaling pathways, such as NF-κB/MAPK (14), Wnt/β-catenin (13), and AMPK pathways (15). Nevertheless, there is no evidence of whether PAA can attenuate renal fibrosis by inhibiting ERS-mediated apoptosis. The aim of this study, therefore, was to investigate the effect of PAA on ERS-induced apoptosis in a model of renal fibrosis.

## Material and Methods

### Chemicals and drugs

PAA (purity ≥98%) was isolated from *Poria cocos* by the method described previously (16). Losartan (LOS), which is a typical ARB, was purchased from Zhejiang HuaHai Pharmaceutical Co., Ltd. (China). Other reagents were of analytical grade.

### Animal experiment

Male Kunming mice aged 6-8 weeks (18-22 g) were purchased from the Animal Centre of Xi'an Jiaotong University (China). Mice were provided with water and standard chow *ad libitum*. All experimental procedures and care of the mice were performed in accordance with the institutional guidelines for animal use in research.

The UUO mouse model was established as the protocol described previously (17). The mice (n=24) for the UUO model were randomly divided into four groups: i) sham-operated group (n=6); ii) UUO group (n=6); iii) UUO+10 mg/kg PAA group (n=6); and iv) UUO+10 mg/kg LOS group (n=6). The mice in the treatment groups were administrated PAA and LOS (10 mg·kg^-1^·day^-1^) by oral gavage for 7 consecutive days after UUO, while the sham operated and UUO groups were administrated an equal volume of normal saline. All the experimental animals were sacrificed at day 7 after UUO.

### Western blot

Total proteins of renal tissues were extracted using RIPA. Protein concentration was detected using BCA Protein Assay Kit (23227, Thermo Scientific, USA), and the protein levels were normalized by α-tubulin expression. Total protein (20-30 μg) was separated by SDS/PAGE and then transferred to polyvinylidene fluoride (PVDF) membranes (10600023, Amersham™ Hybond™, GE Healthcare, USA). PVDF membranes were washed in 1×Tris-buffered saline with 0.1% Tween-20 (TBST) 3 times. After blocking in 5% non-fat milk for 1h, the PVDF membranes were incubated in primary antibody overnight at 4°C. The membranes were washed in TBST 4 times and then incubated in secondary anti-bodies of goat anti-rabbit (1:5000, ab6721; Abcam, USA) or goat anti-mouse (1:5000, A21010; Abbkine, USA) for 2 h. After washing in TBST 6 times, the membranes were visualized using an enhanced chemiluminescence detection reagent (RPN2232, GE Healthcare) and images were acquired by Tanon 6600 Luminescent Imaging Workstation (Tanon Science & Technology Co., Ltd., China). Signal intensities of immunoblots were quantified with ImageJ software (version 1.48 v, NIH, USA) and normalized to the expression levels of α-tubulin (1:1000, 11224-1-AP, Proteintech, China). Quantification of each protein was repeated 3 times. The primary antibodies included: collagen I (1:5000, ab34710, Abcam), fibronectin (1:1000, ab2413, Abcam), vimentin (1:2000, ab92547, Abcam), α smooth muscle actin (α-SMA,1:300, ab7817, Abcam), E-cadherin (1:2000, ab76055, Abcam), CHOP (1:1000, 2895, CST), GRP78 (1:1000, ab108615, Abcam), eIF-2α (1:1000, 9722, CST), P-eIF-2α (1:1000, 9721, CST), ATF4 (1:1000, AF2560, Beyotime), and α-tubulin (Proteintech).

### Quantitative real-time PCR

Total RNA was extracted from kidney with TRIzol reagent (Servicebio, China) according to the manufacturer's instructions. Then, mRNA was used to synthesize cDNA using the cDNA synthesis kit (Servicebio). Quantitative real-time PCR (qRT-PCR) was performed by using SYBR^®^ Premix Ex Taq™ II (Takara Bio, Japan). Forward and reverse primers used in this study are listed in Table 1. After 40 cycles on the PCR machine, the 2^-△△CT^ method was used to calculate the relative expression of mRNA.

**Table 1:** Primers for qRT-PCR.

Genes	Species	Forward	Reverse	Product size (bp)
Collagen I	Mouse	GAGAGGTGAACAAGGTCCCG	AAACCTCTCTCGCCTCTTGC	153
Fibronectin	Mouse	AAGGCTGGATGATGGTGGACT	TCGGTTGTCCTTCTTGCTCC	140
Vimentin	Mouse	TCCAGAGAGAGGAAGCCGAAA	GCAAGGATTCCACTTTCCGTTC	102
*α-SMA*	Mouse	GTACCACCATGTACCCAGGC	GAAGGTAGACAGCGAAGCCA	152
E-cadherin	Mouse	CGACCGGAAGTGACTCGAAAT	TCAGAACCACTGCCCTCGTAAT	188
*Chop*	Mouse	CCAGGAAACGAAGAGGAAGAAT	CACTGACCACTCTGTTTCCGTTT	206
*GRP78*	Mouse	GACGCACTTGGAATGACCCT	TAACCTTCTTTCCCAAATACGCC	198
*ATF4*	Mouse	AGACACCGGCAAGGAGGATG	AAGAGCTCATCTGGCATGGTTT	126
*GAPDH*	Mouse	CCTCGTCCCGTAGACAAAATG	TGAGGTCAATGAAGGGGTCGT	133

### Histological analysis

Kidney tissue was fixed in 4% formaldehyde for 24 h and then embedded in paraffin. Kidney sections (5 μm) were stained with hematoxylin-eosin (HE) and Masson's trichrome regent by a standard protocol (18). Immunohistochemical (IHC) staining was carried out and assessed by routine protocol (19). The images of stained sections were photographed by a LIRI-2006 microscope (Shanghai Optical Instrument Factory, China) and CMOS camera (Shanghai Optical Instrument Factory). Image analysis was performed using Image-Pro Plus 6.0 software. The results of HE staining was assessed by four indicators: tubular epithelial cells loss (score: 0-5), renal tubule dilation (score: 0-5), inflammatory cell infiltration (score: 0-5), and proximal tubule atrophy (score: 0-5). The analysis of Masson's trichrome staining was evaluated by collagen area (score: 0-10) and staining intensity (score: 0-10).

For IHC staining, the results were evaluated by the percentage of positive cells and staining intensity. The score for the percentage of positive cells ranged from 0 to 20 (score=0: negative, score=5: fewer than 10% positive cells, score=10: 10-50% positive cells, score=15: 51-75% positive cells, and score=20: over 75% positive cells). The staining intensity of the positive reaction was classified from 0 to 15 (score=0: colorless, score=5: pale-yellow, score=10: brown-yellow, and score=15: saddle-brown).

### TUNEL staining

TUNEL staining was used to detect tubular cell apoptosis. Briefly, the kidney tissue sections were deparaffinized and incubated with proteinase K (20 mg/L) for 20 min. After washing in phosphate buffered saline (PBS) 3 times, the kidney sections were treated with 3% hydrogen peroxide for 20 min. Then, the sections were washed and incubated with TUNEL reaction mixture containing TDT enzyme-dUTP for 1 h at 37°C. The stained sections were observed and captured under a LIRI-2006 microscope (Shanghai Optical Instrument Factory) and CMOS camera (Shanghai Optical Instrument Factory). TUNEL-positive cells were quantitatively analyzed by two independent pathologists, and the percentage of apoptotic cells was determined.

### Molecular docking and molecular dynamic simulation

The three-dimensional (3D) structure of PAA was collected from PubChem database (https://pubchem.ncbi.nlm.nih.gov/). The crystal structures of GRP78 (PDB: 6asy) and ATF4 (PDB: 1ci6) were collected from RCSD protein data bank (https://www.rcsb.org). The molecular docking experiment was performed using AutoDock 4.2.6 following the standard procedures (20). The result of the interaction between proteins and ligand was visualized by a PyMOL program.

Molecular dynamics simulation (MDS) was performed by Gromacs 2020.6 software combined with Charmm 36 force field. The PAA-GRP78/ATF4 complex was placed in the center of a cubic box filled with TIP3P water. During the MDS process, hydrogen bonds were constrained by the LINCS algorithm with a time step of 2 fs. The electrostatic interaction is calculated by PME method at a distance cut-off of 1.2 nm. We used Berendsen algorithm for NVT equilibration for 100 ps, and set the temperature at 300 K and the pressure at 1 bar. Finally, a 50-ns MDS was carried out. Based on the results, the stabilities of the PAA-GRP78/ATF4 complex were evaluated by root mean square deviation (RMSD), root mean square fluctuation (RMSF), radius of gyration (Rg), and hydrogen bond distribution.

### Statistical analysis

Data are reported as means±SD. Results for multiple groups were analyzed by one-way ANOVA followed by a two-tailed Student's *t*-test between two groups using GraphPad Prism software (GraphPad Software, USA). P<0.05 was considered to be statistically significant.

## Results

### PAA inhibited renal fibrosis and epithelial-to-mesenchymal transition (EMT) in UUO mice

**Figure 2:**
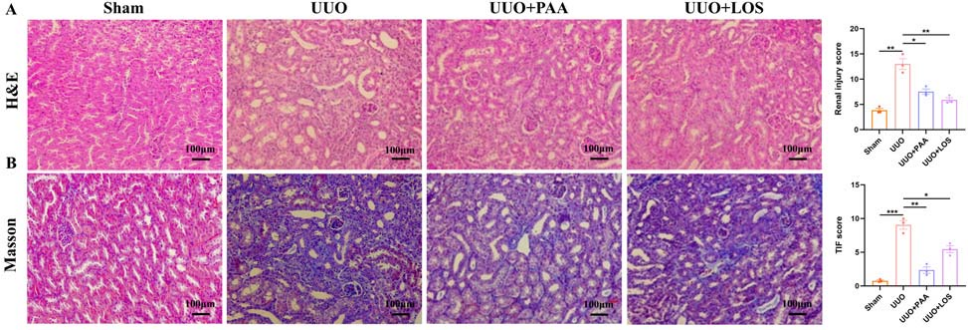
Poricoic acid A (PAA) improved pathologic lesions and collagen deposition in renal unilateral ureteral obstruction (UUO) mice. **A**, HE staining and renal injury score in UUO mice. **B**, Masson's trichrome staining and tubulo-interstitial fibrosis (TIF) score in UUO mice. Scale bar 100 μm. Data are reported as means±SD. *P<0.05, **P<0.01, and ***P<0.001. One-way ANOVA followed by a two-tailed Student's *t*-test. LOS: losartan.

**Figure 3:**
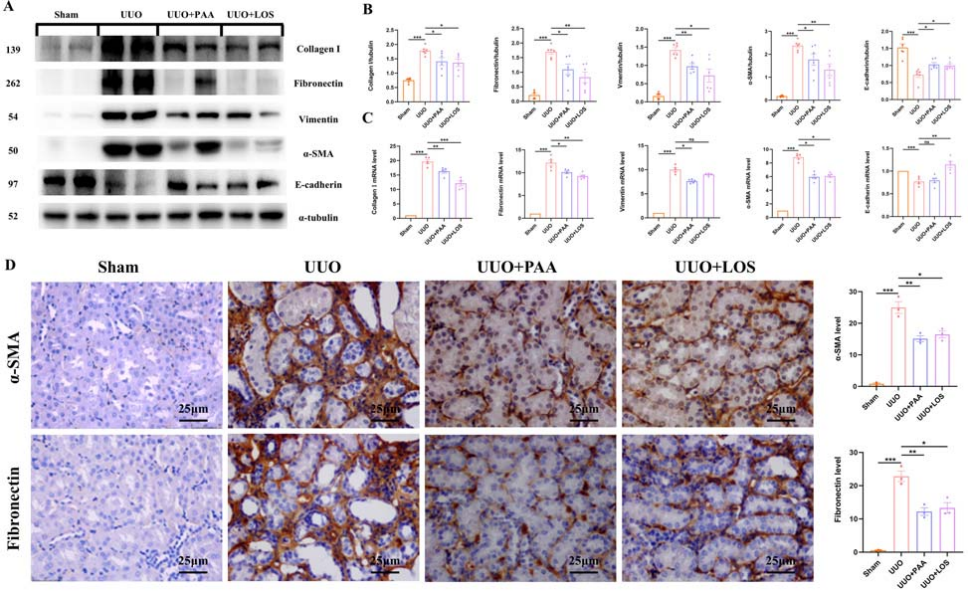
Poricoic acid A (PAA) inhibited epithelial-to-mesenchymal transition (EMT) in the kidney of unilateral ureteral obstruction (UUO) mice. **A** and **B**, Protein expression and quantitative analysis of collagen I, fibronectin, α-SMA, vimentin, and E-cadherin. **C**, mRNA expression of collagen I, fibronectin, α-SMA, vimentin, and E-cadherin. **D**, Immunohistochemical staining of α-SMA and fibronectin and relative quantitative analysis. Scale bar 25 μm. Data are reported as means±SD. *P<0.05, **P<0.01, ***P<0.001, and ns: not statistically significant. One-way ANOVA followed by a two-tailed Student's *t*-test. LOS: losartan

As shown in Figure 2A and B, HE and Masson's trichrome staining indicated that UUO mice had renal injury and tubulo-interstitial fibrosis (TIF). Treatment with PAA significantly attenuated pathological lesions, inflammatory cell infiltration, and collagen deposition. EMT exerts an essential role in the development of renal fibrosis (21). The inhibitory effect of PAA on EMT was examined by measuring the protein and mRNA expression of collagen I, fibronectin, α-SMA, vimentin, and E-cadherin. Compared to the sham-operated group, mice with UUO showed a significant upregulation of collagen I, fibronectin, α-SMA, and vimentin. Following treatment with PAA, the expressions of collagen I, fibronectin, α-SMA, and vimentin were reduced. The protein expression of E-cadherin was increased in the renal tissues of UUO mice and decreased in PAA-treated UUO mice. LOS exhibited similar effect of PAA (Figure 3A-C). Similarly, IHC staining showed the excessive expression of α-SMA and fibronectin in the renal tissues of UUO mice and expression was restored after the treatment of PAA (Figure 3D). Collectively, these findings confirmed that PAA treatment can attenuate renal injury and inhibit EMT.

### PAA inhibited ERS activation in UUO mice

**Figure 4:**
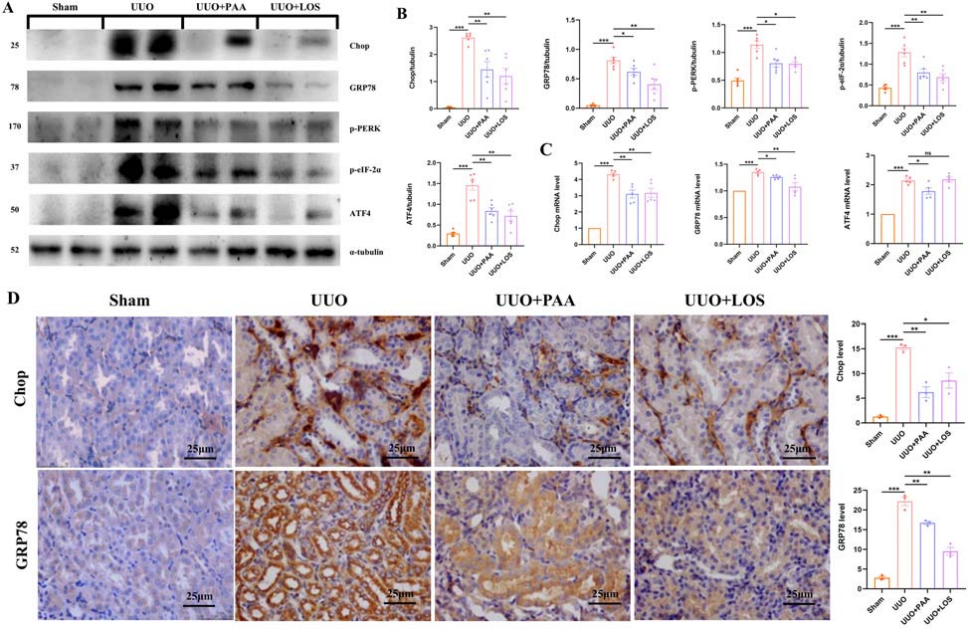
Poricoic acid A (PAA) inhibited endoplasmic reticulum stress (ERS) activation in the kidney of unilateral ureteral obstruction (UUO) mice. **A** and **B**, Protein expression and quantitative analysis of Chop, GRP78, p-PERK, p-eif-2α, and ATF4. **C**, mRNA expression of Chop, GRP78, and ATF4. **D**, Immunohistochemical staining of Chop and GRP78 and relative quantitative analysis. Scale bar 25 μm. Data are reported as means±SD. *P<0.05, **P<0.01, ***P<0.001, and ns: not statistically significant. One-way ANOVA followed by a two-tailed Student's *t*-test. LOS: losartan.

ERS is as a major contributor in the pathogenesis and development of renal fibrosis (8). To investigate the effect of PAA on ERS activation, western blot analysis and qRT-PCR were performed to detect ERS markers, including CHOP, GRP78, p-PERK, p-eif-2α, and ATF4. As shown in Figure 4A-C, the protein and mRNA expression of CHOP, GRP78, p-PERK, p-eif-2α, and ATF4 were markedly increased in UUO mice and significantly decreased after PAA administration. IHC staining showed that the upregulation of CHOP and GRP78 in UUO mice was significantly inhibited after the treatment of PAA (Figure 4D). These results indicated that the effect of PAA against renal fibrosis may be mediated by the repression of ERS activation.

### PAA ameliorated ERS-mediated apoptosis in UUO mice

**Figure 5:**
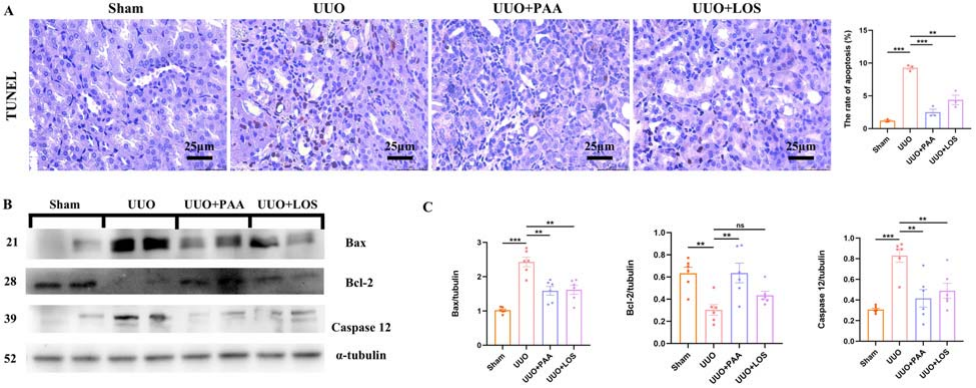
Poricoic acid A (PAA) inhibited cell apoptosis in the kidney of unilateral ureteral obstruction (UUO) mice. **A**, TUNEL staining of the kidney tissues. **B** and **C**, Protein expression and quantitative analysis of Bax, Bcl-2, and caspase 12. Scale bar 25 μm. Data are reported as means±SD. **P<0.01, ***P<0.001, and ns: not statistically significant. One-way ANOVA followed by a two-tailed Student's *t*-test. LOS: losartan.

Cell apoptosis in the kidney tissues of UUO mice was evaluated by TUNEL assay. The number of apoptotic renal tubular epithelial cells in UUO mice was significantly higher than in sham-operated mice, which was significantly decreased by the treatment of PAA (Figure 5A). Western blot and qRT-PCR results showed the increase of pro-apoptoic Bax and caspase 12 expressions, along with the decrease of anti-apoptotic Bcl-2 expression in the renal tissues of UUO mice. The treatment of PAA significantly attenuated the abnormal expression of Bax, Bcl-2, and caspase 12 (Figure 5B). These results demonstrated that PAA might exert a renoprotective function by alleviating ERS-mediated apoptosis.

### Molecular docking and MDS analysis of PAA with GRP78 and ATF4

**Figure 6:**
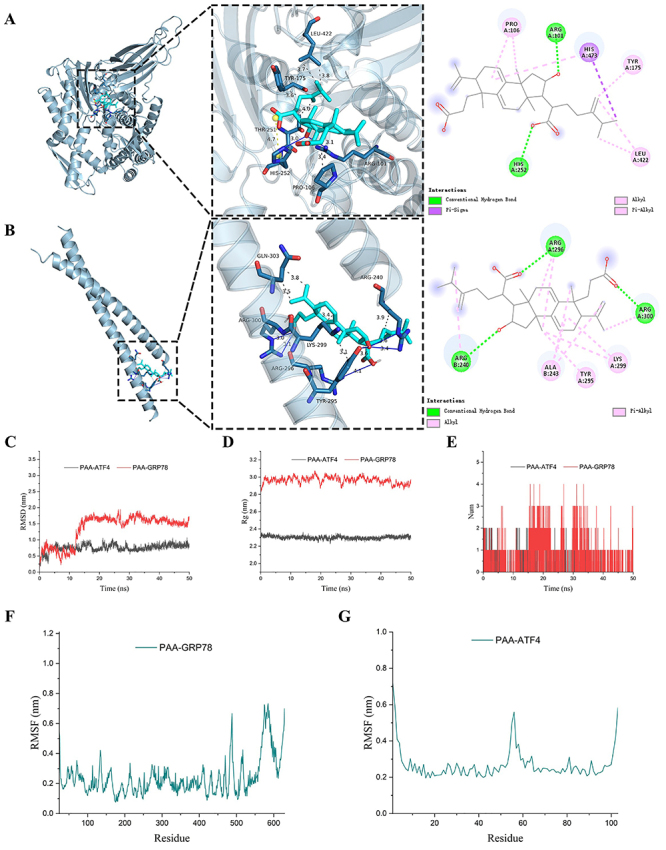
Molecular docking and MDS analysis of poricoic acid A (PAA) with GRP78 (**A**) and ATF4 (**B**). **C**, Root mean square deviation and (**D**) radius of gyration analysis for the PAA-GRP78/ATF4 complex. **E**, Number of hydrogen bonds formed between PAA and GRP78 or ATF4. Root mean square fluctuation analysis for (**F**) PAA-GRP78 complex and (**G**) PAA-ATF4 complex.

To further elucidate the interactions between PAA and ERS-associated proteins, molecular docking was carried out. The affinities of PAA with GRP78 (-6.675 kcal/mol) and ATF4 (-5.133 kcal/mol) were less than -5 kcal/mol, indicating that their combinations were stable. As shown in Figure 6A and B, PAA showed hydrophobic interaction with the amino acid residue ARGA 101 and HISA 252 of GRP78, while forming hydrogen bonds with ARGA 296, ARGA 300, and ARGB 240 of ATF4. Additionally, PAA could bind to GRP78 and ATF4 through different mechanisms, including Alkyl, Pi-Alkyl, and Pi-Sigma.

The structural stability of PAA-GRP78/ATF4 complex was explored by RMSD, RMSF, Rg, and hydrogen bond distribution. As shown in Figure 6C, RMSD of PAA-ATF4 complex fluctuated around 0.75 nm and was stable during the 50 ns MDS. RMSD of PAA-GRP78 complex became stable after 25 ns. Both PAA-ATF4 and PAA-GRP78 formed stable complexes. GRP78 showed a higher average radius and fluctuation, indicating that PAA might have some impact on the compactness of GRP78 (Figure 6D). In addition, the number of hydrogen bonds formed between PAA and GRP78 varied greatly with the simulation time compared with the PAA-ATF4 complex (Figure 6E). Moreover, the majority of the amino acid residues in the binding site showed a small degree of flexibility with a RMSF of less than 0.4 nm (Figure 6F and G), indicating that these domains of proteins have a better binding with PAA.

## Discussion

**Figure 7:**
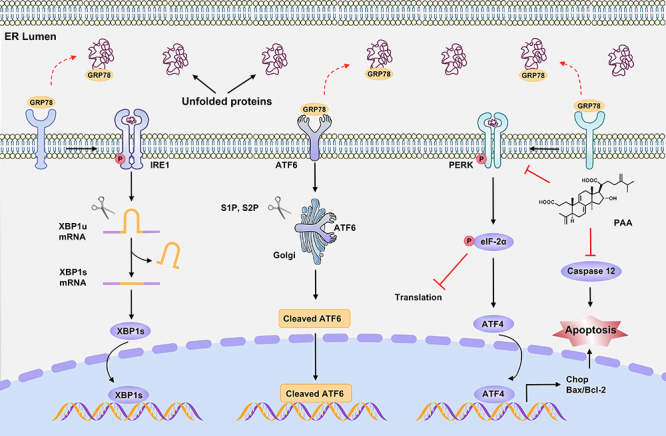
Diagram of the mechanism of poricoic acid A (PAA) against renal fibrosis by inhibiting endoplasmic reticulum stress (ERS)-mediated apoptosis. The underlying mechanism of PAA against renal fibrosis is associated with the inhibition of PERK-eIF2α-ATF4-CHOP signaling pathway and the consequent ERS-associated apoptosis. ER: Endoplasmic reticulum.

The occurrence and development of renal fibrosis resulted in the activation of ERS (22). In the present study, we confirmed that PAA, which is a potent anti-fibrotic small molecule from *Poria cocos*, significantly attenuated renal interstitial fibrosis by inhibiting ERS-mediated apoptosis (Figure 7). Our results showed that the improvement effect of PAA on renal injury in UUO mice was similar to that of LOS, which is the first-line drug for the clinical treatment of CKD. Moreover, PAA significantly reduced the collagen deposition in the renal tissue of UUO mice, which was even more pronounced than LOS. These findings demonstrated the potential of PAA as a novel drug for the treatment of CKD.

UUO induced intrarenal ERS activation and the expression of CHOP, GRP78, eif-2α, p-eif-2α, and ATF4. In the kidney tissues of UUO mice, the activation of ERS was associated with abnormal EMT, as indicated by the upregulation of collagen I, fibronectin, α-SMA, and vimentin and the downregulation of E-cadherin. The treatment of PAA and LOS inhibited the activation of ERS and alleviated aberrant EMT in kidney tissues of UUO mice. Molecular docking and MDS results indicated that PAA formed stable complexes with GRP78 and ATF4, which further indicated that ERS may be the potential signaling pathway for PAA to exert an anti-fibrotic effect.

It is widely accepted that the expression of CHOP is elevated in response to ERS, which can trigger cell apoptosis by inhibiting the expression of Bcl-2 (23). In our study, the western blot and immunohistochemistry results showed that the expression of CHOP was significantly upregulated in the model group, suggesting that the apoptotic pathway of ERS was activated. The results of the TUNEL assay indicated that UUO induced apoptosis of renal tubular epithelial cells in UUO mice, while the treatment of PAA significantly reversed this effect. GRP78 is an ERS-associated hallmark protein and is markedly increased when ERS is activated (24). When ER is under normal physiological conditions, the three sensor factors ATF6, PERK, and IRE1 are inactivated by binding to GRP78. When ERS is activated, the combination of GRP78 and unfolded protein increased, resulting in the dissociation of PERK, I/RE1, and ATF6 from GRP78, which in turn initiates UPR and activates downstream pathways, such as the PERK axis (25,26). Afterwards, the activation of PERK promoted the phosphorylation of eif-2α and enhanced the translation of ATF4. The upregulation of ATF4 induced the expression of CHOP, resulting in cell apoptosis (26,27). In this research, the expression of GRP78, ATF4, p-PERK, and p-eif-2α was markedly increased, which confirmed the previous results, indicating that ERS occurs in UUO mice.

ERS and UPR are considered as a protective mechanism of cells and organisms, while excessive ERS can destroy the homeostasis of UPR, causing inflammation, apoptosis, and oxidative stress (27). Notably, the three pathways of UPR may not be activated simultaneously (28), and the role of PERK-eIF2α-ATF4-CHOP (15) and IRE1α-XBP1s pathways (22) has been widely associated with various diseases. However, this study proved that the effect of PAA against renal fibrosis is only associated with PERK-eIF2α-ATF4-CHOP signaling pathway, which is consistent with the previously reported mechanism of renin in UUO mice (29). Additionally, Chen et al. (30) reported that forsythiaside A can alleviate sepsis-induced acute kidney injury via PERK-eIF2α-ATF4-CHOP pathway-mediated apoptosis and inflammation, which is consistent with our results. These findings demonstrated that ERS exerts an essential role in the occurrence and development of kidney disease through the PERK-eIF2α-ATF4-CHOP signaling pathway.

There is currently a strong body of research indicating that traditional Chinese medicine can prevent many kidney diseases with few side effects (31). An increasing number of prescriptions containing *Poria cocos* have shown renoprotective effects, such as Wulingsan and Jinkuishenqi pills (32,33). Our previous studies also showed that *Poria cocos* epidermis can improve renal function in rats with chronic renal injury, and the mechanism of the renoprotective effect involves fatty acid metabolism, phospholipid metabolism, tryptophan metabolism, and purine metabolism (34). Tetracyclic triterpenoids are considered to be the active chemical components of *Poria cocos*, of which PAA is the major component (34,35). PAA has shown promising pharmacological activities in multiple diseases, such as renal fibrosis (36), diabetic kidney disease (37), myocardial infarction (38), and acute lymphoblastic leukemia (39). Although previous studies have demonstrated that the anti-renal fibrotic effect of PAA is associated with Sirt3/β-catenin (36) and AMPK signaling pathway (15), the relationship between its anti-fibrotic activities and ERS is still unclear.

In this study, we first proved that the development of renal interstitial fibrosis is accompanied by the activation of ERS, and PAA can attenuate renal fibrosis by suppressing the cell apoptosis mediated by the activation of ERS. However, current investigations on the anti-fibrotic activity of PAA mostly focused on cells and animal models, which require further clinical trials to confirm the actual effects on humans. In addition, due to the complexity of disease occurrence and the limitation of research methods, the anti-fibrotic mechanisms of PAA have not been fully revealed. A variety of effective new sequencing techniques, such as metabolomics, transcriptomics, and microbiome, should be applied to further explore the mechanisms of PAA in the future. This direction will provide a solid basis for the clinical application of PAA.

### Conclusion

In summary, this study demonstrated that PAA can alleviate renal fibrosis in UUO mice by inhibiting ERS-mediated apoptosis. Targeting the ERS pathway might provide a potential therapeutic strategy to inhibit the progress of renal interstitial fibrosis and ameliorate kidney function.
